# Empirical and theoretical investigation into the potential impacts of insecticide resistance on the effectiveness of insecticide‐treated bed nets

**DOI:** 10.1111/eva.12574

**Published:** 2017-12-04

**Authors:** Katey D. Glunt, Maureen Coetzee, Silvie Huijben, A. Alphonsine Koffi, Penelope A. Lynch, Raphael N'Guessan, Welbeck A. Oumbouke, Eleanore D. Sternberg, Matthew B. Thomas

**Affiliations:** ^1^ Department of Entomology The Pennsylvania State University University Park PA USA; ^2^ Wits Research Institute for Malaria Faculty of Health Sciences University of the Witwatersrand Johannesburg South Africa; ^3^ ISGlobal Barcelona Ctr. Int. Health Res. (CRESIB) Hospital Clínic—Universitat de Barcelona Barcelona Spain; ^4^ Institut Pierre Richet (IPR) Abidjan Côte d'Ivoire; ^5^ College of Life and Environmental Sciences, Penryn Campus University of Exeter Cornwall UK; ^6^ London School of Hygiene and Tropical Medicine London UK

**Keywords:** *Anopheles*, insecticide resistance, malaria, transmission

## Abstract

In spite of widespread insecticide resistance in vector mosquitoes throughout Africa, there is limited evidence that long‐lasting insecticidal bed nets (LLINs) are failing to protect against malaria. Here, we showed that LLIN contact in the course of host‐seeking resulted in higher mortality of resistant *Anopheles spp*. mosquitoes than predicted from standard laboratory exposures with the same net. We also found that sublethal contact with an LLIN caused a reduction in blood feeding and subsequent host‐seeking success in multiple lines of resistant mosquitoes from the laboratory and the field. Using a transmission model, we showed that when these LLIN‐related lethal and sublethal effects were accrued over mosquito lifetimes, they greatly reduced the impact of resistance on malaria transmission potential under conditions of high net coverage. If coverage falls, the epidemiological impact is far more pronounced. Similarly, if the intensity of resistance intensifies, the loss of malaria control increases nonlinearly. Our findings help explain why insecticide resistance has not yet led to wide‐scale failure of LLINs, but reinforce the call for alternative control tools and informed resistance management strategies.

## 1 INTRODUCTION

About 1 billion long‐lasting insecticidal bed nets (LLINs) have been distributed in Africa in the last 10 years, and these have contributed to substantial declines in the burden of malaria (Bhatt et al., 2015). Over the same period, resistance to pyrethroids has increased dramatically in malaria mosquito vectors (Hemingway et al., 2016; Ranson & Lissenden, 2016). The vast majority of LLINs are treated with pyrethroids alone, and there is now major concern that the rapid spread of this pyrethroid resistance could render LLINs ineffective, compromising not only current control but also potentially undoing the public health gains of recent years (Hemingway et al., 2016). As yet, however, there is no clear indication of wide‐scale control failure of LLINs (Strode, Donegan, Garner, Enayati, & Hemingway, 2014; Thomas & Read, 2016; Viana, Hughes, Matthiopoulos, Ranson, & Ferguson, 2016; World Health Organization 2016a). The reason for this is unclear, but, given the enormous public health implications, better understanding the link (or lack thereof) between resistance and disease control is a major research priority (Sternberg & Thomas, 2017; Thomas & Read, 2016).

The WHO standard bioassay method for monitoring and evaluating insecticide resistance exposes young (<5 days old) female mosquitoes to discriminating doses of insecticide for a fixed time, and mortality is then assessed after 24 hr (World Health Organization 2016b). A vector population is designated insecticide‐resistant when at least 10% of individuals survive the exposure. Although widely used for detecting resistance in field populations, it is now becoming clear that this phenotypic assay tells us little about how resistance interacts with malaria epidemiology (Bradley et al., 2017; Ochomo et al., 2017; Oxborough et al., 2015; Ranson & Lissenden, 2016; World Health Organization 2016a). The reasons for this disconnect could be manifold (Rivero, Vézilier, Weill, Read, & Gandon, 2010). For example, several studies suggest that resistance levels decrease as mosquitoes age (Chouaibou et al., 2012; Jones et al., 2012). Given that malaria parasites take about 2 weeks to develop within the mosquito, transmission might still be halted if older mosquitoes remain susceptible to insecticides, even where younger mosquitoes can survive exposure (Read, Lynch, & Thomas, 2009; Saddler & Koella, 2015). There is also evidence for delayed effects of insecticide exposure leading to reduced daily survival later in life, again potentially impacting the subset of mosquitoes old enough to transmit malaria (Viana et al., 2016). Other studies reveal potential interactions between insecticide resistance and parasite infection and development, which could reduce vectorial capacity of resistant mosquitoes irrespective of mortality (Alout, Djègbè, et al., 2014; Alout, Yameogo et al., 2014).

Here, we add further insights into the complex interactions between insecticide resistance and malaria epidemiology. We first conduct a set of studies to examine both the lethal and sublethal effects of LLIN exposure on a range of resistant mosquito strains from the laboratory. We find that mosquitoes classified as resistant still suffer substantial mortality following exposure to an LLIN, and those that survive suffer reduced blood feeding and host‐searching efficiency for several hours postexposure. We next extend studies to resistant mosquitoes from field populations and largely confirm the empirical results under more natural settings. Finally, we use a model to explore the influence of both sublethal and lethal effects of LLIN exposure on the lifetime transmission potential of resistant mosquitoes. This model indicates that even modest lethal and sublethal effects, when accrued across the lifetime of the mosquito, can have substantial impact on malaria transmission potential, especially under conditions of high LLIN coverage.

## 2 MATERIALS AND METHODS

### 2.1 Mosquito maintenance and strain information

We conducted a range of experiments on resistant *Anopheles* from the laboratory and field. We conducted experiments on a total of five laboratory strains, although because of idiosyncrasies in rearing, not all strains were available in sufficient numbers to be used in all experiments. The laboratory strains of mosquitoes were reared and maintained according to the standard procedures of the Vector Control Research Laboratory (VCRL) in Johannesburg, South Africa, described in Hunt et al. (2005). Strains differed by species and by insecticide resistance selection background: *Anopheles arabiensis*, SENN‐BASE and SENN‐DDT; *An. funestus*, FUMOZ‐BASE and FUMOZ‐R; and *An. gambiae*, TONGS.

SENN‐BASE, originating from Sennar, Sudan, has been maintained at the VCRL since 1990. SENN‐BASE exhibits moderate resistance to pyrethroids only (Oliver & Brooke, 2016). SENN‐DDT was established in 1995 by selecting SENN‐BASE for resistance to DDT: each generation, the survivors of an hour‐long exposure to 4% DDT are allowed to breed and start the next generation (Oliver & Brooke, 2014). SENN‐DDT is resistant to DDT, permethrin, deltamethrin, and malathion due to increased detoxification enzyme activity and fixation of the L1014F *kdr* mutation (Oliver & Brooke, 2013, 2014).

FUMOZ‐BASE and FUMOZ‐R are *An. funestus* strains from southern Mozambique that have been maintained at the VCRL since 2000. Selection of the FUMOZ‐BASE strain with 0.1% lambda‐cyhalothrin, a pyrethroid, from 2000 to 2005 generated the FUMOZ‐R strain, which has increased resistance to pyrethroids and carbamates (Hunt et al., 2005), which is still present in FUMOZ‐BASE (Venter et al., 2017). No *kdr* alleles are present in either strain (Okoye, Brooke, Hunt, & Coetzee, 2007), so the resistance is due to metabolic changes (Amenya et al., 2008; Wondji et al., 2009).

TONGS is an *Anopheles gambiae s.s*. strain colonized in 2010 from mosquitoes collected in Tongon, Côte d'Ivoire. The colony is resistant to pyrethroids, DDT, carbamates, and organophosphates, but the resistance mechanisms remain unidentified (Venter et al., 2017).

We conducted additional laboratory studies on one field strain collected from Palmeíra, Mozambique (25°15′49.5″S, 32°52′13.8″E). On two mornings (between 7 and 11 a.m.), bloodfed female anophelines were collected from human dwellings using mouth aspirators. In the CISM insectary in Manhiça, mosquitoes were provided an oviposition substrate and ad lib access to sugar water for four nights. On the fifth or sixth nights after collection, females were deprived of sugar for about twelve hours before being used in experiments.

These field‐collected females were identified morphologically as *An. funestus*. Due to the nature of field collections, the females were of unknown age and insecticide exposure history. The exact resistance profile was also unknown, although resistance testing of the offspring of females collected in 2013 from the same location using the WHO tube bioassay indicated high‐level resistance to deltamethrin (Glunt et al., 2015), as did CDC bottle bioassays using field‐collected females (5% mortality at diagnostic concentration, 68% mortality at 10X; S Huijben, unpublished data).

We also conducted experimental hut studies at the M'bé field station, near Bouake in central Côte d'Ivoire. The malaria vectors in this location are dominated by *An. gambiae s.s*. (99% M‐form, now classified as *An. coluzzi*) and exhibit intense pyrethroid resistance due to both *kdr* and metabolic mechanisms (Koffi, Ahoua Alou, Adja, Chandre, & Pennetier, 2013; Koffi et al., 2015). CDC assays indicate >1,700‐fold resistance to deltamethrin relative to a standard susceptible strain (see Supporting Information).

### 2.2 Effects of realistic contact with an LLIN on mortality of resistant mosquitoes in the laboratory

The aim of our initial experiment was to examine whether mosquitoes classified as “resistant” still suffer significant mortality following realistic contact with an LLIN. Laboratory studies on the performance of bed nets often involve exposure of adult mosquitoes within a small plastic cone for 3 min, with mortality then assessed after 24 hr (World Health Organization 2016b). This WHO cone test provides a measure of biological efficacy of an LLIN (Koffi et al., 2015; World Health Organization 2016b) and, while not designed for testing resistance, is widely used to explore potential impacts of resistance by comparing efficacy of a given LLIN against resistant and susceptible strains (e.g., Allossogbe et al., 2017; Bagi et al., 2015; Strode et al., 2014; Viana et al., 2016). We used this test to expose four laboratory‐maintained mosquito strains (*Anopheles arabiensis*, SENN‐BASE and SENN‐DDT; *An. funestus*, FUMOZ‐BASE; and *An. gambiae,* TONGS) to a standard LLIN. Five groups of five female mosquitoes were exposed to either untreated netting or pieces of a new Olyset LLIN for 3 min and mortality recorded 24 hr later. A 3‐min exposure of a susceptible laboratory strain against this LLIN prior to use in this study yielded >90% mortality after 24 hr, indicating the LLIN to be effectively impregnated with insecticide (K. Glunt, unpublished data).

We then exposed mosquitoes from these lines to the same LLIN in a more realistic exposure scenario, allowing mosquitoes to search for a human host lying under the net within a large indoor enclosure. We set up either an untreated net or an Olyset LLIN over a twin‐sized air mattress within a larger screen room (1.5 × 1.5 × 2 m) (see Fig. S1). During each trial replicate, a volunteer human host lay on the mattress and we released groups of 50 female mosquitoes into the screen enclosure. Females were recaptured at the end of 1 hr and their mortality recorded 24 hr later. Exposures and assays were conducted during the mosquitoes’ scotophase.

### 2.3 Effects of LLIN exposure on blood feeding

We next examined whether a mosquito that had survived contact with a net would take a blood meal from an accessible host, as might happen if the arm of a host was resting against the side of the net, or if the net was damaged and the mosquito found its way inside. We used three laboratory‐maintained mosquito strains and WHO tubes fully lined with LLIN (a new PermaNet 2.0 LLIN), or untreated netting for controls to ensure a forced exposure. The shift in brand of LLIN between assays was necessitated by unforeseen competing demand for nets from other experiments (see later discussion for possible implications). Mosquitoes were exposed for durations that varied with the intensity of resistance of each strain (i.e., 1 min for the least resistant, *An. arabiensis*/SENN‐DDT; 5 min for the intermediate strain, *An. funestus*/FUMOZ‐BASE; and 10 min for the most resistant strain, *An. funestus*/FUMOZ‐R). This variation in exposure duration was designed to generate some level of mortality following exposure, but with sufficient numbers of mosquitoes surviving to enable subsequent testing. The increasing exposure time assumed that mosquitoes with more intense resistance might spend longer searching around an LLIN before being impacted by lethal or sublethal effects (as we later discuss, however, the time different mosquitoes spend in contact with an LLIN is very poorly characterized). A 3‐min exposure of a fully susceptible laboratory strain to the LLIN generated 100% mortality (Fig. S2), indicating that the net was effectively treated (World Health Organization 2016b).

We used 10 mosquitoes per tube with three replicate tubes. Immediately following exposure, mosquitoes were transferred to mesh‐covered cups and the arm of a human host (KDG) placed directly on the mesh. After five minutes, we counted the number of mosquitoes with any amount of blood in their abdomen. Exposures and assays were conducted during the mosquitoes’ scotophase under dim white light. Sugar was provided 1 hr postexposure and mortality assessed after 24 hr.

### 2.4 Effects of LLIN exposure on host‐seeking

We also examined whether there were any impacts of LLIN exposure on host‐seeking behavior. We exposed two resistant laboratory strains in netting‐lined WHO tubes as described above, as well as the field‐collected strain from Mozambique. We added about 20 females to each WHO tube for these host‐seeking assay exposures, with three replicate tubes. Individual females were transferred to a small mosquito cage (BugDorm; 14 × 15 × 15 cm) by mouth aspirator. The human host (KDG) pressed a forearm against the left side of the cage and started a stopwatch after releasing the mosquito and exhaling into the cage once from the front. Exhalations were repeated every 30 s to encourage host searching. If the mosquito did not respond, the test terminated after 2 min. If a female probed through the mesh on either side with host cues (i.e., the front and left sides), the test terminated, and this was recorded as “time to host.” Assays were carried out at various times post‐LLIN exposure (i.e., approximately 1, 7, and 24 hr) to characterize the time course of any behavioral changes. Assays started a minimum of 15 min postexposure, and trials alternated between females exposed to untreated netting and LLIN. Only females able to fly were selected. Host‐seeking assays were conducted under red light.

### 2.5 Impacts of LLIN exposure on survival and feeding under field conditions

To complement the laboratory‐based experiments, we extended our investigations to the field, using experimental huts in an area of known pyrethroid resistance in central Côte d'Ivoire (Koffi et al., 2013, 2015) to examine the effectiveness of a standard LLIN (PermaNet 2.0) against naturally recruiting wild mosquitoes. Huts were the typical West African experimental hut design (World Health Organization 2013) with LLIN and untreated control nets damaged according to standard protocols (Koffi et al., 2015; World Health Organization 2013) to enable mosquitoes to take blood meals. Adult male volunteers (informed consent according to ethical approval #022/MSLS/CNER‐dka provided by Le Comite National d'Ethique de la Recherche of Côte d'Ivoire) slept in the huts each night between 19:00 and 05:00. At 05:00, volunteers dropped a sheet between the hut and the veranda and then collected all mosquitoes from both locations. Mosquitoes were taken to the laboratory, scored for blood feeding and mortality, and identified to species complex. Huts were cleaned daily and the nets rotated between huts over five replicate nights.

### 2.6 Mathematical model to explore the implications for malaria transmission potential

To evaluate the consequences of the lethal and sublethal effects revealed in the empirical studies for overall transmission, we developed a deterministic feeding‐cycle model. The model was similar to others previously used to evaluate transmission‐related metrics for malaria vectors (e.g., Cator, Lynch, Thomas, & Read, 2014; Waite, Lynch, & Thomas, 2016). The structure and details are summarized in the Supplementary Information, with parameter estimates given in Table S2. Due to the paucity of available field data for certain parameters, a wide range of parameter values was explored in a sensitivity analyses (see Supporting Information). The results were generated using a version of the model executed using Mathcad.

The model was used to calculate the average number of infectious bites per mosquito lifetime for various assumptions regarding bed net coverage and per‐feeding‐attempt levels of LLIN‐associated mosquito mortality and feeding inhibition. We used this to calculate values for relative transmission potential (RTP), which we define as the average number of infectious bites across the lifetime of a vector relative to the number of infectious bites if there was no LLIN‐related mortality or feeding impairment. Thus, if RTP = 0, transmission is completely halted, whereas RTP = 1 is equivalent to baseline transmission in the absence of LLINs. If the rate of recruitment to the adult vector population and the size of the human population are both assumed to be unaffected by an intervention, RTP can be assumed to map directly to a proportional change in the entomological inoculation rate (EIR) (Waite et al., 2016).

### 2.7 Statistical analysis

We analyzed the effects of treatments on survival using binomial generalized linear models, employing the quasibinomial distribution as necessary to correct for overdispersion (indicated in results by *F*‐statistics instead of chi‐square). To compare proportions (e.g., cone assay to net assay outcomes), we used the prop.test function in R (version 3.2.1). To evaluate the effect of exposure on the time taken to locate the host, individual female flight times and responses were used to run Cox proportional hazard models, with timepoint postexposure and treatment as factors. Mean time to host and the standard error of the mean (SEM) were calculated by averaging the flight duration of all responding females from each treatment group.

## 3 RESULTS

### 3.1 Effects of realistic contact with an LLIN on mortality of resistant mosquitoes in the laboratory

**Figure 1:**
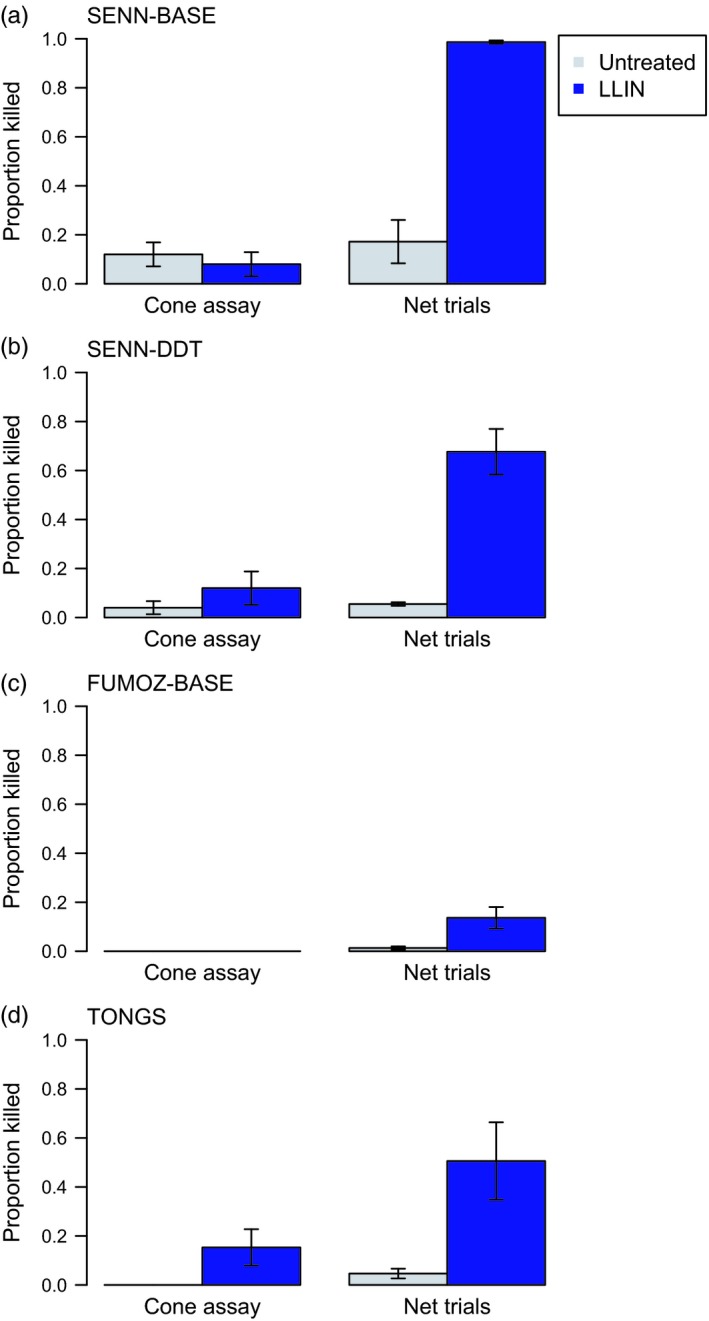
Mortality of mosquito strains exposed to an long‐lasting insecticidal bed net (LLIN) in cone assays or in a free‐ranging laboratory trials. When five groups of five females were exposed to an Olyset LLIN for 3 min in WHO cone assays, few were killed. When fifty females were released into a 1.5 × 1.5 × 2 m enclosure and allowed to interact freely with the LLIN placed over a human host, we observed significantly greater mortality (*p *<* *.05). This was true regardless of *Anopheles* species or strain: (a) and (b) *An. arabiensis*, (c) *An. funestus*, (d) *An. gambiae*. Bars show mean values ± *SEM*. Gray bars show mortality from exposures to an untreated net, while the blue bars show mortality for an LLIN

The standard WHO cone assays yielded mortality ranging from 0% to 20%, which did not differ significantly from equivalent exposure to an untreated net (Fig. 1; *An. arabiensis*/SENN‐BASE: *N*_untreated_ = 25, *N*_LLIN_ = 25, χ^2^ = 0, *p *=* *1; SENN‐DDT: *N*_untreated_ = 49, *N*_LLIN_ = 50, χ^2^ = 1.2, *p *=* *.3; *An. funestus*/FUMOZ‐BASE: *N*_untreated_ = 45, *N*_LLIN_ = 44, no mortality in either group, so test not possible; *An. gambiae*/TONGS: *N*_untreated_ = 24, *N*_LLIN_ = 26, χ^2^ = 2.2, *p *=* *.1).

Assays with mosquitoes free‐flying around a host protected by the same LLIN resulted in 17%–98% mortality, compared with 4%–20% following equivalent exposure to an untreated net (Fig. 1). LLIN‐induced mortality was significantly higher than the cone assays for all strains tested (SENN‐BASE: *N*_untreated_ = 144, *N*_LLIN_ = 146, χ^2^ = 133.2, *p *<* *.001; SENN‐DDT: *N*_untreated_ = 146, *N*_LLIN_ = 285, χ^2^ = 51.7, *p *<* *.001; FUMOZ‐BASE: *N*_untreated_ = 152, *N*_LLIN_ = 181, χ^2^ = 5.2, *p *=* *.02; TONGS: *N*_untreated_ = 196, *N*_LLIN_ = 149, χ^2^ = 9.6, *p *=* *.002).

### 3.2 Effects of LLIN exposure on blood feeding

**Figure 2:**
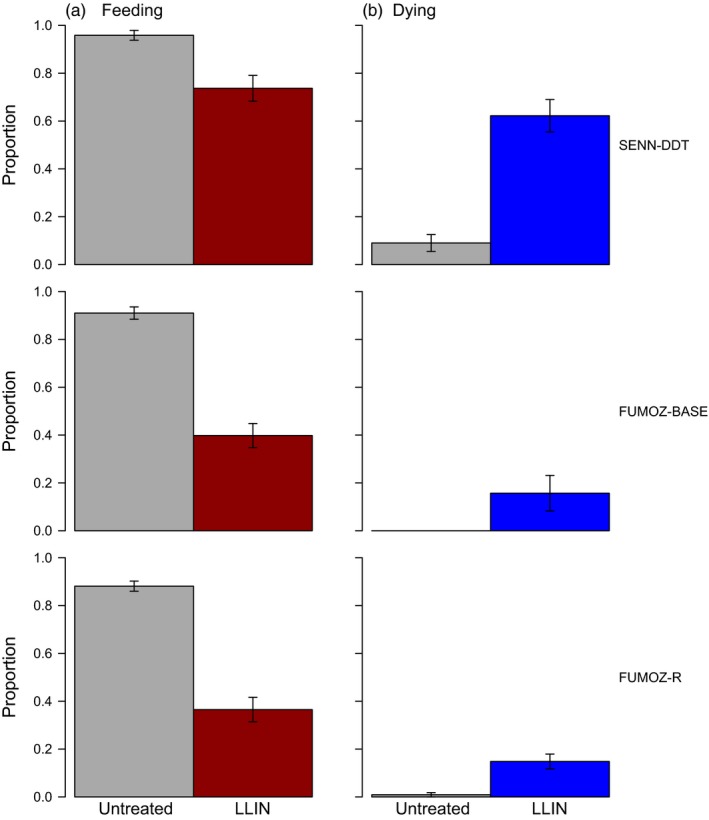
Blood feeding success and mortality following long‐lasting insecticidal bed net (LLIN) exposure. Two groups of ten mosquitoes were exposed to untreated netting or PermaNet 2.0 for 1 min (SENN‐DDT), 5 min (FUMOZ‐BASE), or 10 min (FUMOZ‐R). (a) Immediately after LLIN exposure, mosquitoes were offered access to a host for 5 min. LLIN‐exposed mosquitoes were significantly less likely to take a blood meal. (b) Survival was assessed 24 hr later. Bars show mean values ± *SEM*; all comparisons significantly different, *p *<* *.05. Gray bars show results for an untreated net, and the colored bars (red for feeding and blue for mortality) show results for the LLIN

In all three mosquito strains, LLIN exposure significantly inhibited blood feeding relative to mosquitoes exposed to an untreated net (Fig. 2A. SENN‐DDT: 139 of 145 fed when exposed to untreated, 105 of 143 fed with the LLIN, *F*_1,27_ = 17.5; FUMOZ‐BASE: 111 of 123 fed with the untreated net, 57 of 139 for the LLIN, *F*_1,23_ = 71.3, FUMOZ‐R: 146 of 166 fed with the untreated net, 61 of 165 with the LLIN, *F*_1,31_ = 78.8; all strains: *p *<* *.001). In the two most resistant strains (the FUMOZ lines), LLIN exposures reduced feeding by approximately 60%, even though mortality after 24 hr was only 15%–20% (Fig. 2b).

### 3.3 Effects of LLIN exposure on host‐seeking

**Figure 3:**
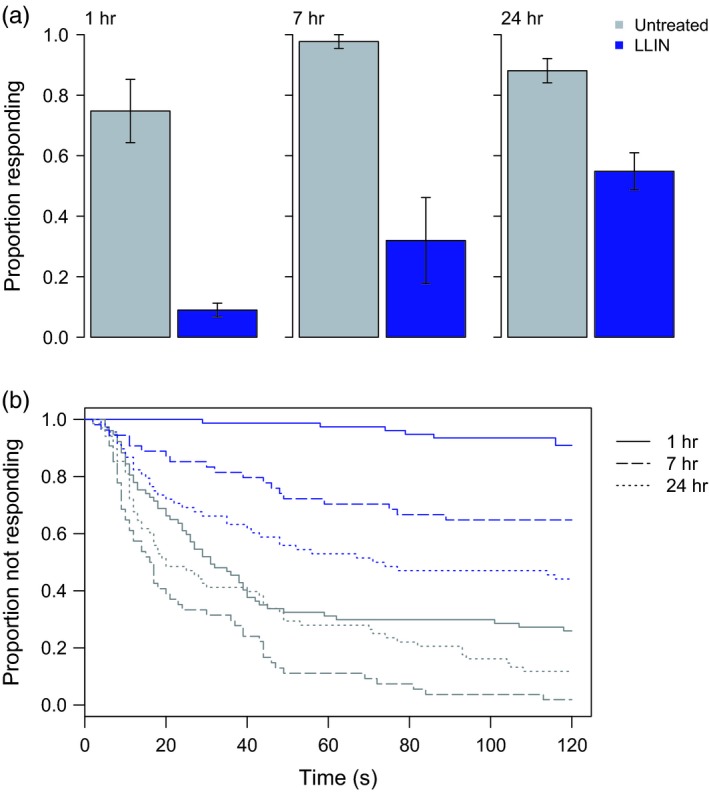
Host‐seeking in resistant *An. arabiensis* (SENN‐DDT) exposed to untreated netting or an long‐lasting insecticidal bed net (LLIN). At about one, seven, or 24 hr after exposure to untreated netting or PermaNet 2.0, individual females were tested for their ability to locate a host over a short distance (~0.15 m) within two minutes. Bars in (a) show mean values ± *SEM*. Shortly after LLIN exposure, SENN‐DDT females were (a) less likely to find a host and those that found the host (b) did so more slowly. By 24 hr after exposure, exposed females were still less likely to find a host, but those able to locate a host were just as fast as unexposed females. Gray bars/lines show results for an untreated net, and the blue bars/lines show results for the LLIN

Resistant *An. arabiensis* (SENN‐DDT) showed a 90% reduction in host response around 1 hr after exposure to an LLIN, relative to mosquitoes exposed to an untreated net (both treatments, *N*_1hr_ = 77). At 7 and 24 hr after exposure (both treatments, *N*_7hr_ = 54, *N*_24hr_ = 68), the relative reductions had lessened to 60% and 30%, respectively (Fig. 3a; GLM; net treatment: $\chi_{1,396}^{2}$ = 124.1, *p* < .001; time postexposure: $\chi_{1,395}^{2}$ = 33.0, p < .001; interaction: $\chi_{1,394}^{2}$ = 3.3, *p* = .07). There was also a significant increase in time to response relative to controls immediately postexposure (Fig. 3b; Cox PH; 1 hr: *z* = 6.7, *p* < .001; 7 hr: *z* = 6.9, *p* < .001; 24 hr: *z* = 3.9, *p* < .001).

**Figure 4:**
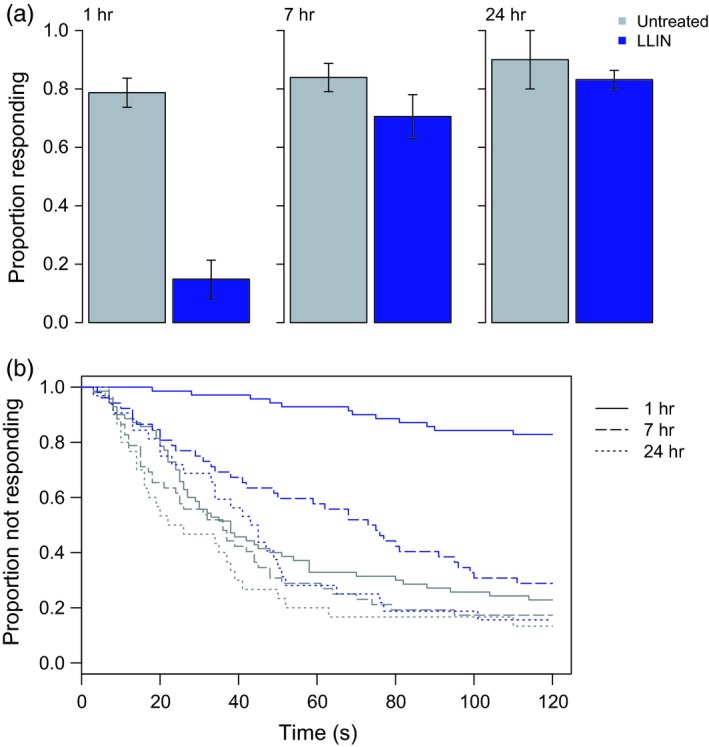
Host‐seeking in resistant *An. funestus* (FUMOZ‐R) exposed to untreated netting or an long‐lasting insecticidal bed net (LLIN). At about one, seven, or 24 hr after exposure to untreated netting or PermaNet 2.0, individual females were tested for their ability to locate a host over a short distance (~0.15 m) within two minutes. Bars in (a) show mean values ± *SEM*. Immediately after LLIN exposure, FUMOZ‐R females were (a) less likely to find a host and those that found the host (b) did so more slowly. Exposed females recovered their short‐range host location ability with seven hours of exposure, though they remained slower than unexposed females for up to 24 hr. Gray bars/lines show results for an untreated net, and the blue bars/lines show results for the LLIN

Exposure of resistant *An. funestus* (FUMOZ‐R) to an LLIN resulted in similar changes in mosquito host response ~1 hr after exposure, with 80% fewer responders and a significant increase in response time (Fig. 4; both treatments, *N*_1hr_ = 70). The percent responders was also lower and the response time greater after 7 hr postexposure (both treatments, *N*_7hr_ = 52; untreated netting *N*_24hr_ = 30, LLIN *N*_24hr_ = 32), although the effects were no longer significant (Fig. 4a; GLM; net treatment: $\chi_{1,304}^{2}$ = 34.5, *p* < .001; time postexposure: $\chi_{1,303}^{2}$ = 33.9, *p* < .001; interaction: $\chi_{1,302}^{2}$ = 9.1, *p* = .003; Fig. 4b. Cox PH; 1 hr: *z* = 6.6, *p* < .001; 7 hr: *z* = 2.4, *p* = .02; 24 hr: *z* = 1.2, *p* = .2).

**Figure 5:**
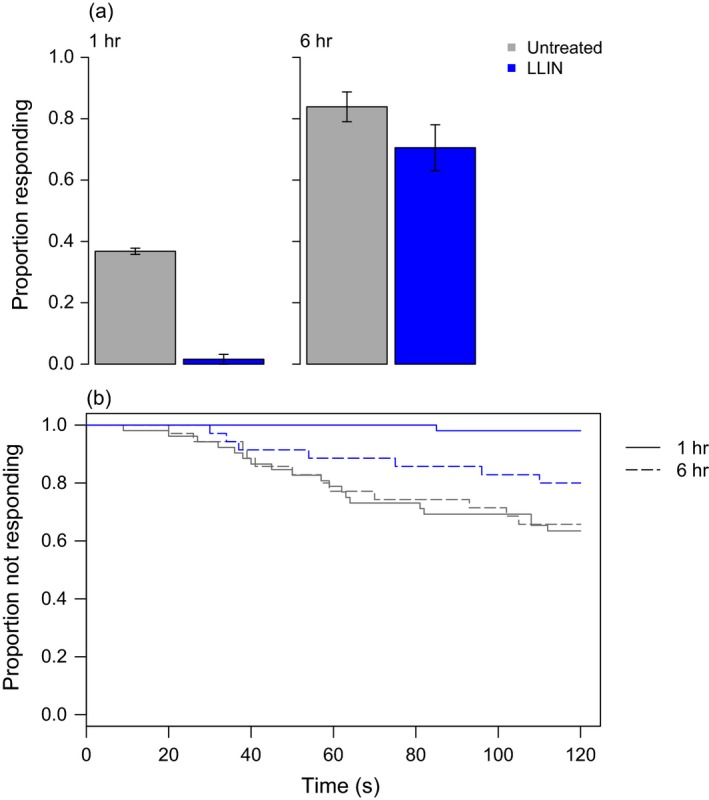
Host‐seeking in field‐caught, resistant *Anopheles* spp. from Mozambique exposed to untreated netting or an long‐lasting insecticidal bed net (LLIN). At about one or six hours after exposure to untreated netting or PermaNet 2.0, individual females were tested for their ability to locate a host over a short distance (~0.15 m) within two minutes. Bars in (a) show mean values ± *SEM*. Immediately after LLIN exposure, field‐caught females were (a) less likely to find a host and those that found the host (b) seemed to do so more slowly, though only one LLIN‐exposed female responded to host cues. Exposed females recovered their short‐range host location ability with seven hours of exposure, although they remained slower than unexposed females. Gray bars/lines show results for an untreated net, and the blue bars/lines show results for the LLIN

Similar results were obtained with field‐caught *An. funestus* from a site of known pyrethroid resistance in Palmeíra, Mozambique (Fig. 5). While overall levels of response were lower with these field mosquitoes, there was again a substantial change in host response behavior around 1 hr after exposure to an LLIN, with 95% fewer responders and a significant increase in response time relative to controls (Untreated, *N*_1hr_ = 52, 6 hr: *N*_6hr_ = 35; LLIN, *N*_1hr_ = 51, *N*_6hr_ = 35). These effects waned by 6 hr postexposure (Fig. 5a. Net treatment, $\chi_{1,171}^{2}$ = 18.1, *p* < .001; time postexposure, $\chi_{1,170}^{2}$ = 1.6, *p* = .2; interaction $\chi_{1,169}^{2}$ = 6.8, *p* = .01; Fig. 5b. Cox PH; 1 hr: *z* = 3.1, *p* = .002; 6 hr: *z* = 1.3, *p* = .2).

### 3.4 Impacts of LLIN exposure on survival and feeding under field conditions

**Figure 6:**
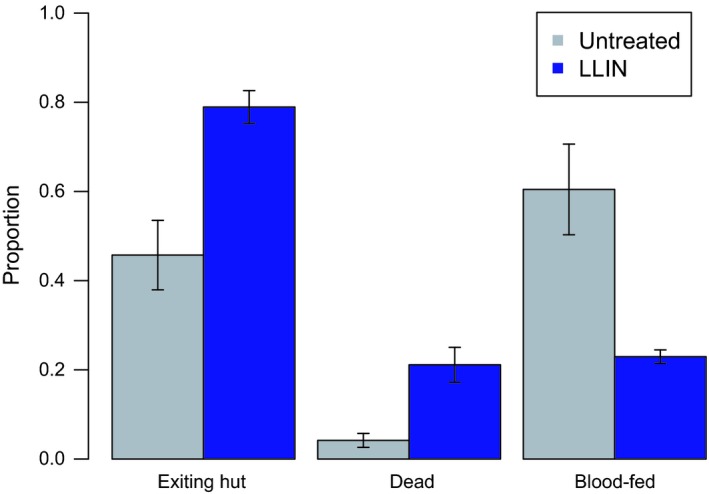
Experimental hut trial in an area with resistant *An. gambiae* outside of Bouake, Côte d'Ivoire. Experimental huts were outfitted with an artificially damaged untreated net or long‐lasting insecticidal bed net (LLIN) (PermaNet 2.0). On five consecutive mornings, mosquitoes were collected and their location and blood feeding status recorded. Mortality was recorded 24 hr later. Bars represent mean values ± *SEM* for the proportion of mosquitoes collected attempting to exit the hut, dead within the hut, and/or with a blood meal. Proportions do not total to one, as the categories are not mutually exclusive. Females that entered a hut with an LLIN were more likely to be found in the veranda of the hut (i.e., exiting the hut) than inside the hut. They were also more likely to be killed, but less likely to take a blood meal (*p *<* *.05). Gray bars show results for an untreated net, and the blue bars show results for the LLIN

Over five nights, 342 female *An. gambiae* were caught in huts with untreated nets, and 387 in huts with LLINs. We found that, in spite of high levels of resistance (Table S1), the LLIN caused an increase in mosquito mortality (Fig. 6. 20% vs. 5%; *F*_1,9_ = 18.6, *p *=* *.003), an increase in hut exit rate (80% vs. 47%; *F*_1,9_ = 17.1, *p *=* *.003), and a reduction in blood feeding (24% vs. 60%; *F*_1,9_ = 13.1, *p *=* *.007), relative to an untreated net. These results suggest the effects we observe in the laboratory assays are broadly observable in field settings.

### 3.5 Implications for malaria transmission potential

Our empirical data indicated that mosquitoes classified as resistant using standard WHO procedures still suffered elevated mortality when trying to contact a host protected by an LLIN and that mosquitoes that survived exposure suffered sublethal effects that inhibited feeding and host searching.

**Figure 7:**
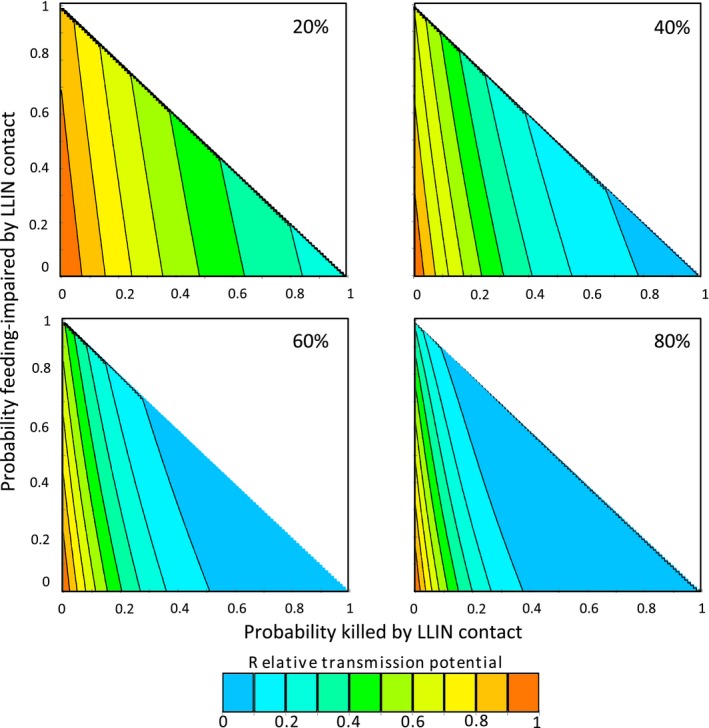
Changes in relative transmission potential for combinations of assumed probabilities of long‐lasting insecticidal bed net (LLIN)‐generated mortality and feeding impairment per feeding attempt. Panels show the relative transmission potential (RTP) of a mosquito population exposed to various levels of LLIN coverage, compared to the RTP if no bed nets were present. Axes represent the probability, during each feeding attempt on an LLIN‐protected host, of prebite mortality (*x*‐axis) or feeding impairment (*y*‐axis) caused by the LLIN. Proportion killed plus proportion impaired cannot exceed 100%, so plots are only generated in range *x *+ *y* ≤ 1

In Fig. 7, we present outputs from the model showing the effects of different levels of mortality and feeding deterrence per feeding attempt on RTP, for various levels of LLIN coverage. At high coverage, we found that resistance (defined in terms of the proportion of mosquitoes that survived an encounter with an LLIN) must reach very high levels before control is substantially affected (i.e., before the RTP of a mosquito population increases above 10%). When coverage is at least 60%, control is sustained at this level as long as half of the mosquitoes that contact an LLIN are killed. Even if resistance was such that only 20% of mosquitoes died following contact with an LLIN, RTP would still be substantially reduced (to 20%–30%) under conditions of high coverage, and this reduction is enhanced if exposure impairs feeding. On the other hand, under conditions of low LLIN coverage, the effects of resistance on RTP are much more marked. Similarly, as resistance increases to very high levels, there is a progressive impact on RTP leading to accelerating control failure. These results are robust across a range of parameter values (see Supporting Information).

## 4 DISCUSSION

Malaria epidemiology is the outcome of complex interactions among a multiplicity of factors, and so, assessing the contribution of any one factor to changes in disease prevalence is difficult (Kleinschmidt et al., 2015). However, given the importance of LLINs (Bhatt et al., 2015), it is critical that we better understand how insecticide resistance might impact control both now and in the future.

Here, we examined how host searching influences the LLIN‐associated mortality of putatively resistant *Anopheles spp*. mosquitoes and, conversely, how LLIN exposure affects the blood feeding success of host‐seeking, insecticide‐resistant mosquitoes. Our data suggest that the WHO characterization of resistance provides little insight into the functional impact of an LLIN. This disconnect could be explained in part by LLIN contact time. Recent video analysis (Parker et al., 2015) of host‐seeking behavior of a susceptible *An. gambiae* strain found that average contact time with an LLIN ranged from 17 to 95 s, versus 80 to 334 s with an untreated net. In both cases, the majority of contact occurred during the first 10 min, with very little active search behavior after 30 min. Other observational studies suggest contact times of up to 7 min on an LLIN (Siegert, Walker, & Miller, 2009) and up to 50 min on an untreated net (Diop et al., 2015). These patterns potentially explain why we saw higher mortality when mosquitoes interacted with an LLIN naturally than in 3‐min WHO cone tests. There could also be an interaction between insecticide exposure and the energetics of host searching. How long resistant mosquitoes spend in contact with an LLIN under field conditions, and whether this changes with intensity or mechanism of resistance, is an important area for further research.

We observed effects of LLIN exposure on host‐seeking and blood feeding in *Anopheles* species that exhibit different mechanisms and intensities of resistance. In most cases, the effects waned between 2 and 7 hr, though the effects lasted for at least 24 hr in one strain (SENN‐DDT). These sublethal effects likely contribute to the ongoing effectiveness of LLINs, even where direct mortality begins to decline. If mosquitoes fail to take a blood meal or suffer disrupted host searching such that they are less likely to feed during a night following LLIN exposure (and so will not get deflected to an unprotected host in a different or even the same house), transmission will be affected.

Our host‐seeking assays evaluated behavioral responses over short distances only and for just a short time. However, other studies that have used similar assays indicate equivalent responses between short‐range/short‐term assays and longer‐range/longer‐term assays (Cator et al., 2013; George et al., 2011). Moreover, if mosquitoes failed to respond to the very pronounced host cues of heat, CO_2_ and other volatiles over a short range, we consider it unlikely that they would respond to more diffuse cues over longer ranges. Siegert et al. (2009) observed that transient contact with an LLIN could result in “disengagement” of host searching by a susceptible strain of *An. gambiae*, possibly due to loss of ability to sense host cues. These results are consistent with our findings, although the disengagement mechanism(s) remain unclear.

The experimental hut studies conducted in Côte d'Ivoire largely corroborate our observations from the laboratory. Numerous other studies have also shown that LLINs continue to have some impact in experimental huts (Churcher, Lissenden, Griffin, Worrall, & Ranson, 2016; Koffi et al., 2015; Strode et al., 2014). What is notable about our finding is that in an area with mosquitoes exhibiting >1,700‐fold resistance to deltamethrin (Table S1), a damaged deltamethrin‐treated LLIN caused significant increases in mortality and repellency, and a significant decrease in blood feeding, relative to an equivalent untreated net. These results suggest that even intense resistance does not render LLINs totally ineffective.

Our transmission model enabled us to explore the consequences of resistance at the community level. The model revealed three key results. First, when lethal and sublethal effects of LLIN exposure are compounded across the lifetime of a mosquito, LLINs can still contribute substantially to reductions in transmission even if resistance reduces their instantaneous effectiveness. At high coverage, for example, RTP remains below 10% whether an LLIN kills 95% of mosquitoes on contact, or just 50%. With sublethal effects (e.g., 30% reduction in feeding success), mortality as low as 30%—levels of the same order as our empirical data—still does not precipitate substantial changes in relative control at the community level. Second, while control is relatively insensitive to the levels of resistance observed in our experiments, progressive reductions in LLIN efficacy (and, in particular, mortality) lead to nonlinear increases in control failure. This observation supports the notion of a “tipping point” (World Health Organization 2012), where further increases in the intensity of resistance could render LLINs ineffective. Alternatively, decay in the insecticide concentration as nets age could interact with resistance to accelerate control failure. Such an effect could have important policy implications as LLINs are meant to last for 3 years and 20 washes, yet resistance could reduce this effective lifespan. Third, the overall impact of resistance is very sensitive to LLIN coverage. High levels of coverage improve control and help buffer initial impacts of resistance, although they also create a tension in that high levels of coverage are likely to maximize selection for increased resistance.

A key model assumption is that mosquitoes suffer equivalent mortality and/or feeding inhibition at each exposure to an LLIN, such that impacts accrue over time. This assumption is supported by empirical data for the FUMOZ‐BASE mosquito line, which shows contact with an LLIN to cause the same (or even increasing) mortality during sequential exposures (Fig. S2). Our model is conservative in other assumptions, as it did not include increases in susceptibility to insecticides as mosquitoes age (Hodjati & Curtis, 1999; Jones et al., 2012), or additional delayed mortality following exposure (Viana et al., 2016). Such factors would further reduce the ability of mosquitoes to transmit malaria and contribute to preserving the efficacy of LLINs in the presence of apparent resistance.

The lethal and sublethal effects following exposure to LLINs that mitigate the anticipated effects of resistance are consistent across multiple species of resistant anophelines, including laboratory and field strains. Some of the laboratory strains (SENN‐DDT, FUMOZ‐R) were selected for enhanced resistance in the laboratory environment, and there is the possibility that laboratory‐based selection results in different resistance mechanisms than natural selection in the field (Crow, 1957; Roush & McKenzie, 1987). However, other laboratory strains (SENN‐BASE, FUMOZ‐BASE) were derived from the field and have not been subject to artificial selection. Moreover, the mosquitoes from Mozambique and Côte d'Ivoire were completely wild.

We were obliged to use a different net type in the initial mortality assay than we used in the other experiments. However, there is little reason to think that the nature of the net affected our overall conclusions. The Olyset LLIN is impregnated with permethrin while the PermaNet 2.0 is coated with deltamethrin, but both pyrethroid insecticides are affected by the common target site and metabolic mechanisms of resistance (Brogdon & McAllister, 1998). Moreover, several studies show continued impact of Olyset nets in experimental hut studies in spite of strong resistance characterized using standard laboratory exposures (Churcher et al., 2016; Strode et al., 2014). For example, a recent experimental hut study conducted in an area of >100‐fold permethrin resistance in Benin showed an Olyset LLIN to cause an average of 32% mortality, compared with 5% mortality using an untreated control net (Ngufor et al., 2016). Further, relative to huts with an untreated net, the LLIN increased the proportion of mosquitoes exiting the huts by 19% and reduced blood feeding by 66% (Ngufor et al., 2016). These results are broadly consistent with our experimental hut studies conducted using PermaNet 2.0.

Our results are in line with those of a recent multicountry epidemiological evaluation, which found no evidence of an association between resistance (measured through standard WHO laboratory bioassays) and malaria prevalence or incidence, but did find significantly lower rates of infection in individuals using LLINs, indicating continued personal protection (Bradley et al., 2017; Ochomo et al., 2017; World Health Organization 2016a). The pyrethroid susceptibility recorded in the multicountry evaluation was suggestive of moderate resistance. Under conditions of high bed net coverage, we predict the epidemiological signal of such resistance to be relatively weak, and this might be especially so with respect to malaria prevalence, which itself exhibits a strongly nonlinear, saturating relationship with measures of transmission intensity (Smith & McKenzie, 2004). This result is consistent with another recent theoretical analysis that suggests negligible increases in transmission intensity (force of infection) when the frequency of insecticide resistance is <40%–50% (although this result is sensitive to LLIN coverage and baseline transmission intensity) (Churcher et al., 2016).

While it is encouraging that the effectiveness of LLINs appears resilient to the onset of insecticide resistance, these findings should not be interpreted as saying that resistance is unimportant. Even if increases in force of infection are small and hence difficult to detect, they still represent an increased risk of transmission. Moreover, further intensification of resistance could well lead to accelerating control failure, especially in areas of low effective LLIN coverage, or as nets age and become damaged and lose active ingredient (see also Churcher et al., 2016). Development of effective insecticide resistance management strategies requires a better understanding of how insecticide resistance affects ultimate disease transmission across different transmission settings (Sternberg & Thomas, 2017).

## AUTHOR CONTRIBUTIONS

MBT and KDG wrote manuscript. KDG, MBT, RN, and AAK designed experiments. KDG, SH, EDS, and AWO performed experiments. KDG analyzed data. Model and modeling by PAL. All authors read and approved final manuscript. No competing interests.

## DATA ARCHIVING STATEMENT

Data available from the Dryad Digital Repository: https://doi.org/10.5061/dryad.vj78t.
