# Supplementary material for: Empirical and theoretical investigation into the potential impacts of insecticide resistance on the effectiveness of insecticide‐treated bed nets
Source: Evol Appl. 2017 Dec 4;11(4):431–41. doi: 10.1111/eva.12574 (PMC5891045; doi:10.1111/eva.12574)
Supplement: Supplementary file 1 [file EVA-11-431-s001.docx]

**Supplementary information:**

Empirical and theoretical investigation into the potential impacts of insecticide resistance on the effectiveness of insecticide-treated bed nets

Katey D. Glunt, Maureen Coetzee, Silvie Huijben, A. Alphonsine Koffi, Penelope A. Lynch, Raphael N’Guessan, Welbeck Achille Oumbouke, Eleanore D. Sternberg, Matthew B. Thomas

Table S1. Deltamethrin resistance in wild *Anopheles gambiae s.s.*, Côte d’Ivoire

Table S2. Baseline parameter values for model

Fig. S1. Diagrams illustrating set up of laboratory experiments.

Fig. S2. Mortality of susceptible mosquito strain.

Fig. S3. Mortality of females exposed to LLIN on successive days.

Model information

*Schematic*

*Assumptions*

*Description*

*Details*

*Sensitivity analysis*

**Table S1.** Resistance to deltamethrin in wild populations of *Anopheles gambiae s.s.* at the experimental hut field site in M’bé, Bouake, Côte d’Ivoire, determined using a CDC bottle bioassay.

| **Strain** | **Slope** (SE) | **LD_50_** (95% CI) | **RR_50_** (95% CI) |
| --- | --- | --- | --- |
| KISUMU | 1.3 (0.18) | 0.0145 (0.0087 - 0.022) |  |
| M’bé | 1.9 (0.23) | 27.88 (19.18 - 33.62) | 1778 (1348.34 - 2345.53) |

LD50 expressed in µg/mL.

Mosquitoes for the CDC bottle assay were collected from the field site as late instar larvae or pupae and reared through to adult under standard insectary conditions. To determine resistance intensity, a range of deltamethrin concentrations (microgram a.i. per 250 ml bottle) that produced mortality between 0-100% were prepared and tested against susceptible (Kisumu) and resistant (M’bé) mosquitoes. At testing, adult mosquitoes were 3-4 days old, with 100 females on average (4 replicates of 25 females) per concentration. The exposure period was 1h, with mortality scored after 24h. The resistance ratio was calculated by dividing the LD_50_ of the resistant strain by that of the susceptible laboratory strain (KISUMU).

**Table S2.** Baseline model parameter values used to investigate how the probability of mortality and blood-feeding impairment following LLIN exposure interact to influence transmission potential

| **Model parameter** | **Description of parameter** | **Value** |
| --- | --- | --- |
| *µ* | Daily average background mortality rate | 0.1 per day^1,2^ |
| *G* | Average length of gonotrophic cycle | 3 days^1^ |
| *c* | Probability of mosquito infection following infected blood meal | 0.022^1,2^ |
| *j* | Extrinsic incubation period of parasite | 9 days^1^ |
| *P_r_* | Probability repelled by LLIN before affected by insecticide | 0.1^3^ |
| *q* | Average search time to locate new host after being repelled by LLIN (without impairment) | 1 hour  (0.042 days) ^2,4^ |
| *q_m_* | Maximum search time before feeding delayed to next night | 0.25 days^2,4^ |
| *M_F1_* & *M_F2_* | Feeding-related mortality (without intervention) | 10% per feed^1,2^,  split evenly between pre- and post-bite |

1. Estimated within biologically plausible range of values (see for example Killeen et al. 2000; Killeen and Smith 2007)

2. Sensitivity analysis (See SI) indicates that the value selected for this parameter is not critical to our conclusions.

3. While there are some data regarding the probability of deflection away from an LLIN, it is not clear what proportion of observed deflection might represent feeding impaired mosquitoes. We have explored the importance of this assumption in the sensitivity analysis (Supplementary information).

4. Considered a reasonable assumption with little field data available.

**Fig. S1. Diagrams showing set up of laboratory experiments 1, 2 and 3.**

**Fig. S2. Mortality of susceptible mosquito strain.**

###

### Five replicates of five *Anopheles arabiensis* (KGB) females were exposed to Permanet 2.0 for 3 minutes, using the WHO cone bioassay (World Health Organization 2016b). Twenty-four hours after exposure, all LLIN-exposed females were dead (25 of 25) and all females exposed to untreated netting were alive (24 of 24); this supports the classification of this strain as “fully susceptible” and that the LLIN was treated with insecticide.

**Fig. S3. Mortality of females exposed to LLIN on successive days**.

On day 1, groups of around 20 female *Anopheles funestus* (FUMOZ-BASE) mosquitoes were exposed to either untreated netting or Permanet 2.0 in WHO bioassay tubes for 5 minutes. Sugar was provided and mortality assessed 24 hours later. After mortality was recorded, surviving individuals were pooled and randomly divided into groups of around 20 females and exposed again for 5 minutes to their respective treatment. This was repeated a third day, to generate sample sizes of 264, 161, 122 exposed to untreated netting and 619, 397, 238 females exposed to LLIN on day 1, 2, and 3, respectively. LLIN-exposed mosquitoes were more likely to die on the second or third days of exposure (*P* < 0.05, generalized linear models); i.e. exposure did not remove more susceptible individuals in a way that reduced the probability of the remaining females dying following subsequent contacts with an LLIN. Mean values ± standard error of the mean.

**Model information**

*Model schematic*

This schematic depicts the possible paths of a mosquito through one feeding cycle and provides a basic visualization of how the mathematical model is set up.

### *Model Assumptions*

1. Vector death occurs as a random event at a constant rate
2. Mosquito mortality rates, deflection and infection probabilities are not affected by mosquito age
3. Infectious vectors remain infectious until death

# *Description of model parameters*

| *Variable* | Description | Units |
| --- | --- | --- |
| *G* | average number of days per gonotrophic cycle | days |
| *j* | extrinsic incubation period | days |
| *P_LP_* | proportion of hosts under LLINs | proportion |
| *P_r_* | probability mosquito approaching host under LLIN is repelled without impairment | probability |
| *P_D_* | probability mosquito is feeding-impaired by LLIN per feeding attempt on LLIN-protected host | probability |
| *M_n1_* | probability of pre-bite mortality caused by LLIN per feeding attempt on LLIN-protected host | probability of death |
| *M_F1_* | pre-bite feeding-related mortality (no intervention) per feeding attempt | probability of death |
| *M_F2_* | post-bite feeding-related mortality (no intervention) per feeding attempt | probability of death |
| *c* | probability mosquito infected when feeding on human host | probability |
| *μ* | average background mortality between feeding attempts | instantaneous daily mortality rate |
| *q* | average time taken to locate a new host after being deflected from a LLIN (unimpaired) | days |
| *q_m_* | maximum total search time before feeding attempt delayed to following night | days |
| *q_n_* | maximum number of (unimpaired) deflections in one night before feeding postponed to following night | deflections |
| *M_r_* | mortality associated with being repelled from a host before attempting to feed | probability of death |
| *P_R_* | probability on a given night that host-seeking mosquito will postpone feeding attempt to following night as a result of non-impairing deflection | probability per night |
| *P_LR_* | probability on a given night that host-seeking mosquito will ultimately attempt to feed on a LLIN-protected host | probability per night |
| *P_NR_* | probability on a given night that host-seeking mosquito will ultimately attempt to feed in property without LLINs | probability per night |
| *P_d_* | probability feeding event results in postponement of feeding attempt to next night (from repeated deflection and/or impairment) | probability per feeding event |
| *P_f_* | probability host seeking event leads to a successful bite (same night) | probability per night |
| *F_L_* | probability that a successfully fed mosquito took its feed on a LLIN protected host | probability per successful feed |
| *S_f_* | probability survives from successful bite to host seek at start of next cycle | probability per bite |
| *S_d_* | probability survives from disrupted feeding attempt to start host-seeking on following night | probability |
| *S_I_* | probability infected mosquito will survive to attempt an infectious feed | probability |
| *P_fA_* | average probability that a host-seeking mosquito will survive to take a feed (whether during this event or postponed because feeding impaired) | probability |
| *B_A_* | average number of subsequent bites which a host seeking mosquito will survive to give | bites |
| *P_I_* | probability vector will acquire a Plasmodium infection during its lifetime | probability |

### *Model Details*

$$P_{D}+M_{n1}\leq1$$

Maximum (unimpaired) deflections in one night before feeding postponed to following night, $q_{n}=\left\lfloor\frac{q_{m}}{q}\right\rfloor$ (equation S1)

Mortality associated with being repelled from a host, $M_{r}=1-e^{-q\mu}$

Probability on a given night that host-seeking mosquito will postpone feeding attempt to following night as a result of non-impairing deflection

$P_{R}=\left(P_{LP}P_{r}\right)^{q_{n}+1}\left(1-M_{r}\right)^{q_{n}}$ (equation S2)

Probability on a given night that host-seeking mosquito will ultimately attempt to feed on a LLIN-protected host, $P_{LR}$ equals $\begin{matrix} P_{LP}\left(1-P_{r}\right)+P_{LP}P_{r}\left(1-M_{r}\right)P_{LP}\left(1-P_{r}\right)+\left(P_{LP}P_{r}\left(1-M_{r}\right)\right)^{2}P_{LP}\left(1-P_{r}\right)+ \\ \text{ }...+\left(P_{LP}P_{r}\left(1-M_{r}\right)\right)^{q_{n}}P_{LP}\left(1-P_{r}\right) \end{matrix}$

which, using standard equations can be written as

$P_{LR}=P_{LP}\left(1-P_{r}\right)\left(\frac{1-\left(P_{LP}P_{r}\left(1-M_{r}\right)\right)^{q_{n}+1}}{1-P_{LP}P_{r}\left(1-M_{r}\right)}\right)$ (equation S3)

Probability on a given night that host-seeking mosquito will ultimately attempt to feed in property without LLINs,$P_{NR}$ equals $1-P_{LP}+P_{LP}P_{r}\left(1-M_{r}\right)\left(1-P_{LP}\right)+\left(P_{LP}P_{r}\left(1-M_{r}\right)\right)^{2}\left(1-P_{LP}\right)+...+\left(P_{LP}P_{r}\left(1-M_{r}\right)\right)^{q_{n}}\left(1-P_{LP}\right)$

which, using standard equations can be written as

$P_{NR}=\left(1-P_{LP}\right)\frac{1-\left(P_{LP}P_{r}\left(1-M_{r}\right)\right)^{q_{n}+1}}{1-P_{LP}P_{r}\left(1-M_{r}\right)}$ (equation S4)

Probability feeding event results in postponement of feeding attempt to next night (from repeated deflection and/or impairment) $P_{d}=P_{LR}P_{D}+P_{R}$

Probability host seeking event leads to a successful bite (same night) $P_{f}=\left(P_{LR}\left(1-M_{n1}-P_{D}\right)+P_{NR}\right)\left(1-M_{F1}\right)$ (equation S5)

Probability that a successfully fed mosquito took its feed on a LLIN protected host, $F_{L}=\frac{P_{LR}\left(1-M_{n1}-P_{D}\right)}{P_{NR}+P_{LR}\left(1-M_{n1}-P_{D}\right)}$ (equation S6)

Probability survives from successful bite to host seek at start of next cycle,

$S_{f}=\left(1-M_{F2}\right)e^{-\mu G}$ (equation S7)

Probability survives from disrupted feeding attempt to start host-seeking on following night,

$S_{d}=e^{-\mu}$ (equation S8)

The probability that an infected mosquito will survive to attempt an infectious feed, $S_{I}$, is

[Probability survives period of one gonotrophic cycle after being infected] x [Probability survives remaining time to infectiousness] x [Probability survives any remaining time until next feeding attempt]

This is influenced by the extent to which it experiences deflection/impairment which results in postponement of feeding attempt to next night (hereafter called ‘delays’), during feeding attempts between infection and infectiousness, since this will affect both specific mortality probabilities and the overall time taken to the first post-infectious host-seeking event.

A newly infected mosquito will next attempt to feed after completing one cycle, with $j-G$ days remaining before its infection becomes infectious. It can then potentially experience repeated delays prior to infectiousness, up to a maximum of one per day for the remainder of the EIP. Its first infectious host-seeking event will be the first following infectiousness, with timings depending on when during a gonotrophic cycle infectiousness is reached.

The probability of surviving from the first host seeking event post infection to the first bite post infection is therefore the sum, for all possible numbers of delays (i.e. 0 to *j-G*), of the probability of surviving both that number of delays and the corresponding number of completed feeding cycles, multiplied by the probability of experiencing that combination of delays and completed feeding cycles. The number of feeding cycles associated with a given number of delays is equal to the number of days between infection and infectiousness minus the days taken to complete the first post infection cycle, minus the number of delays, divided by the length of a gonotrophic cycle, rounded up to the nearest whole number.

The probability that an infected mosquito will experience a given number of delays and completed feeding cycles between infection and infectiousness is the probability of experiencing that combination × the number of orders in which that combination can be experienced.

If the total number of days for a combination of delays and corresponding completed feeding cycles is greater than the number of days between infection and infectiousness, then the sequence must be terminated by a completed feeding cycle, so the number of orders in which that sequence can be experienced is the number of possible orders of delay and feeding cycle between the first and last completed feeding cycles. If the number of days between infection and infectiousness is equal to the total days in a given combination of delays and completed feeding cycles, then all events after the first completed feeding cycle can be in any order. The total number of orders in which *r* out of *n* objects can be arranged is given by the binomial coefficient $\frac{n!}{\left(n-r\right)!r!}$.

The probability that a newly infected mosquito will survive to host-seek as an infectious individual is therefore;

(equation S9)

Note that equation S9 uses ‘$\left\lceil x\right\rceil$’ notation for the mathematical ceiling function, which gives the least integer greater than or equal to $x$, with negative values rounded up towards 0.

Average probability that a host-seeking mosquito will survive to take a feed (whether during this event or postponed because feeding impaired), $P_{fA}$, equals $P_{f}+P_{d}S_{d}P_{f}+\left(P_{d}S_{d}\right)^{2}P_{f}+...+\left(P_{d}S_{d}\right)^{\infty}P_{f}$ which, using standard equations can be written as

$P_{fA}=\frac{P_{f}}{1-P_{d}S_{d}}$. (equation S10)

Average number of subsequent bites which a host seeking mosquito will survive to give, $B_{A}$, equals $P_{fA}+P_{fA}S_{d}P_{fA}+\left(S_{d}P_{fA}\right)^{2}P_{fA}+...+\left(S_{d}P_{fA}\right)^{\infty}P_{fA}$ which, using standard equations can be written as

$B_{A}=\frac{P_{fA}}{\left(1-S_{f}P_{fA}\right)}$ (equation S11)

Probability vector will acquire a Plasmodium infection during its lifetime, $P_{I}$ equals $P_{fA}c+P_{fA}\left(1-c\right)S_{f}P_{fA}c+\left(P_{fA}\left(1-c\right)S_{f}\right)^{2}P_{fA}c+...+\left(P_{fA}\left(1-c\right)S_{f}\right)^{\infty}P_{fA}c$ which, using standard equations can be written as

$P_{I}=\frac{P_{fA}c}{\left(1-P_{fA}S_{f}\left(1-c\right)\right)}$ (equation S12)

Vector average lifetime infectious bites,

$V_{LAIB}=P_{I}S_{I}B_{A}$ (equation S13)

*Model Sensitivity Analysis*

1. The significance of **LLIN coverage** is illustrated in figure 7 of the main text. The impact of LLIN-associated mortality and feeding impairment varies with LLIN coverage, so we have used one of the intermediate values as a base for the sensitivity analysis, all figures assume 60% LLIN coverage.

2. **Sensitivity to *μ*, mosquito natural mortality rate**

Base value = 10% per day mortality

Lower background mortality increases the impact of LLIN related mortality on vector lifetime infectious bites relative to the value with no intervention (RTP). Remaining lethal effects and feeding impairment continue to show benefits from LLINs in the presence of resistance, in accord with our conclusions, with assumed daily mortality rates above and below the rate used for the analysis in the main text.

3. **Sensitivity to *G,* average length of gonotrophic cycle.**

Base value = 3 days

In general, assuming a shorter duration for the gonotrophic cycles increases the impact of mortality and feeding impairment on RTP, since it increases the number of potential exposures of infected vectors to all sources of feeding related mortality. However, it is also the case that changing the assumed feeding cycle length generates a noticeably non-linear relationship between the probability of feeding impairment and the RTP. This arises because the parasite extrinsic incubation period (EIP) is not a multiple of the feeding cycle length. Introducing a given delay between feeding cycles during the EIP can therefore result in the first infectious bite being attempted sooner than would be the case without the delay. For example, a 10-day EIP with 3-day feeding cycles would mean the first infectious bite would be 12 days after infection. However, if feeding impairment delayed a feeding attempt by 1 day during the EIP, the first infectious bite might be ten days after infection.

4. **Sensitivity to c, probability vector acquires *Plasmodium* infection per human blood meal.**

Base value = 2.2%

RTP simply scales with *c*, so selected value has no impact on results.

5. **Sensitivity to *j*, length of extrinsic incubation period in days**

Base value = 9 days

In general, assuming a longer duration for the extrinsic incubation period (EIP) increases the impact of LLIN-related mortality and feeding impairment on RTP, since it increases the number of potential exposures of infected vectors to all sources of feeding-attempt-related mortality before infectiousness is reached. However, it is also the case that assuming an EIP that is not a multiple of the feeding cycle length generates a noticeably non-linear relationship between the probability of feeding impairment and the RTP, for the reasons previously explained in the notes regarding the gonotrophic cycle length sensitivity above. With no feeding impairment, the time to first infectious feeding attempt for a given EIP is the lowest multiple of the gonotrophic cycle length equal to or greater than the EIP. Hence, the results along the x-axis, with no feeding impairment, are the same for either 8 or 9 day EIP. This relationship is lost when impairment can add additional single days to the time between infection and the first post-infectious feeding attempt.

6. **Sensitivity to *P_r_,*, probability repelled by LLIN (unimpaired) before attempting to feed on LLIN protected host**

Base value = 10% (LLIN coverage at 60%)

LLIN coverage at 80%

The probability of being repelled before experiencing impairment or mortality effects of LLINs predictably reduces the impact of LLIN-related mortality and feeding impairment on RTP. This has a quantitative, though not qualitative effect on our results. This effect is minimal with high levels of LLIN use.

7. **Sensitivity to *q,*, average time required to locate a new host after (unimpaired) deflection away from a LLIN protected host**

Base value = 1 hour

The value assumed for this parameter has minimal impact on our results.

8. **Sensitivity to *q_n_*, average cumulative search time before (unimpaired) deflected vector abandons host seeking until the following night**

Base value = 6 hours

The value assumed for this parameter has minimal impact on our results.

9. **Sensitivity to *M_F1_* & *M_F2_*, pre- and post-bite feeding-related mortality (not from LLIN)**

Base value = 10% mortality split evenly pre- and post-bite

The assumed background feeding related mortality affects our results quantitatively but not qualitatively.

References:

Killeen, G. F., McKenzie, F. E., Foy, B. D., Schieffelin, C., Billingsley, P. F., & Beier, J. C. (2000). A simplified model for predicting malaria entomologic inoculation rates based on entomologic and parasitologic parameters relevant to control. *American Journal of Tropical Medicine and Hygiene*, *62*, 535–544. <https://doi.org/10.4269/ajtmh.2000.62.535>

Killeen, G. F., & Smith, T. A. (2007). Exploring the contributions of bed nets, cattle, insecticides and excitorepellency to malaria control: A deterministic model of mosquito host-seeking

behaviour and mortality. *Transactions of the Royal Society of Tropical Medicine and Hygiene*, *101*, 867–880. https://doi.org/10.1016/j.trstmh.2007.04.022
